# Role of Animal Models to Advance Research of Bacterial Osteomyelitis

**DOI:** 10.3389/fvets.2022.879630

**Published:** 2022-04-26

**Authors:** Caroline Billings, David E. Anderson

**Affiliations:** Large Animal Clinical Sciences, University of Tennessee College of Veterinary Medicine, Knoxville, TN, United States

**Keywords:** osteomyelitis, bone, *in vivo*, animal model, *Staphylococcus aureus*

## Abstract

Osteomyelitis is an inflammatory bone disease typically caused by infectious microorganisms, often bacteria, which causes progressive bone destruction and loss. The most common bacteria associated with chronic osteomyelitis is *Staphylococcus aureus*. The incidence of osteomyelitis in the United States is estimated to be upwards of 50,000 cases annually and places a significant burden upon the healthcare system. There are three general categories of osteomyelitis: hematogenous; secondary to spread from a contiguous focus of infection, often from trauma or implanted medical devices and materials; and secondary to vascular disease, often a result of diabetic foot ulcers. Independent of the route of infection, osteomyelitis is often challenging to diagnose and treat, and the effect on the patient's quality of life is significant. Therapy for osteomyelitis varies based on category and clinical variables in each case. Therapeutic strategies are typically reliant upon protracted antimicrobial therapy and surgical interventions. Therapy is most successful when intensive and initiated early, although infection may recur months to years later. Also, treatment is accompanied by risks such as systemic toxicity, selection for antimicrobial drug resistance from prolonged antimicrobial use, and loss of form or function of the affected area due to radical surgical debridement or implant removal. The challenges of diagnosis and successful treatment, as well as the negative impacts on patient's quality of life, exemplify the need for improved strategies to combat bacterial osteomyelitis. There are many *in vitro* and *in vivo* investigations aimed toward better understanding of the pathophysiology of bacterial osteomyelitis, as well as improved diagnostic and therapeutic strategies. Here, we review the role of animal models utilized for the study of bacterial osteomyelitis and their critically important role in understanding and improving the management of bacterial osteomyelitis.

## Introduction: Clinical Disease and Patient Impact

Osteomyelitis is an inflammatory bone disease that results in progressive bone destruction and bone loss and is typically caused by infectious microorganisms (1–4). The most common causative organisms are bacteria (1), specifically Gram-positive Staphylococci such as *Staphylococcus aureus* (*S. aureus*) (1–3, 5–8). There are three main etiologies of osteomyelitis: hematogenous, trauma or surgery associated, and secondary to vascular disease. Hematogenous osteomyelitis is most common among pediatric patients (5, 7, 8). Injury associated osteomyelitis may be spread from a contiguous focus of infection, may be secondary to trauma, or may be associated with surgery, especially where implanted medical devices are used. This may occur in individuals of any age (2, 3, 5–7). Osteomyelitis also commonly occurs secondary to vascular insufficiency and is often a result of diabetic foot ulcers (DFU) (2, 6, 7). The annual incidence rate of osteomyelitis in the United States is not precisely known. In 1999, the incidence was reported to be as high as one out of every 675 hospital admissions, which translates to approximately 50,000 cases annually (9). Since that time, the incidence of osteomyelitis cases of all categories has been increasing (8). The rise in caseload is partially due to increases in cases of diabetes (8), trauma (10), numbers of reconstructive orthopedic procedures and implanted prosthetic materials (6, 11–13), and also may be associated with improvements in diagnosis (2).

Clinical presentation of patients suffering from osteomyelitis is variable. Acute osteomyelitis may present with fever, redness, pain and draining lesions. Symptoms of chronic osteomyelitis may be vague, with a wide array of clinical features which may be as subtle as simple focal swelling and tenderness on physical examination (2, 6, 7, 11, 14, 15). Nonspecific clinical presentation necessitates a thorough patient workup for successful diagnosis (16). Diagnostic testing often includes physical examination, hematology and biochemistry panels, measurement of C-reactive protein (CRP), culture and sensitivity testing of bone and wound samples, and imaging such as radiographs and ultrasound. Radiographic evidence of boney changes lag behind pathologic changes, so early disease may not be apparent on standard radiographs (7, 11, 17). Advanced imaging can be helpful, and magnetic resonance imaging (MRI) or computed tomography (CT) (1, 11, 16) may be required. Despite the abundance of available tests that may be employed, there are few early pathognomonic findings for osteomyelitis (7, 18, 19). Therefore, while osteomyelitis may be suspected, the gold standard of diagnosis requires a bone biopsy for culture (2, 5, 11, 18–20) and histopathologic examination (7, 11, 18–21).

Osteomyelitis results in significant morbidity and mortality to the patient (6, 10, 14, 22, 23), and expedient, intensive treatment is indicated. The most common clinical approach to treatment of bacterial osteomyelitis involves a combination of medical and surgical management (4, 6, 11, 18, 24, 25). Systemic antibiotic therapy should be guided by microbial cultures whenever possible (11). In the absence of culture and sensitivity results, empirical, broad-spectrum antibiotics are usually administered (4, 5, 18). Antimicrobial therapy is typically administered for a minimum of 4–6 weeks (2, 19, 20, 24, 26) and is often continued for longer periods of time in an attempt to mitigate risks of chronic osteomyelitis (20). Some clinicians advocate treatment for up to six months after diagnosis (19, 20, 26). Local antibiotic therapy may be instituted to complement systemic antimicrobial therapy (27). Surgical debridement of affected tissue is routine treatment in conjunction with medical management (2, 15, 18, 19). A hallmark of osteomyelitis is the presence of necrotic bone (2, 6, 15), which is readily colonized and surrounded by biofilm (11, 28). Biofilms often result in persistence of bacterial infection. Persistence is multifactorial and is partially due to the protective slime matrix that provides a physical barrier between immune cells and bacterial cells (6, 11, 14) and can impair diffusion of antibacterial substances (29). Persistence also results from the physiologic environment of biofilms, which allows for enhanced antimicrobial resistance through creation and persistence of immense phenotypic diversity, including metabolically inactive bacteria and subpopulations of “persisters” or phenotypically resistant bacteria (30–33). Debridement of necrotic bone should be thorough, with the goal of reaching healthy, viable tissue and removing sources of biofilm. This often includes removing implanted hardware (2, 5). While this approach sounds straightforward and reasonable to accomplish, there are many challenges in the treatment of osteomyelitis which often leave patients suffering relapses or struggling with chronic infections (11, 34–36). Particular challenges include inadequate debridement (2, 15, 19, 30), metabolically inactive bacteria or bacteria embedded in biofilm (2, 12, 30), inadequate antimicrobial penetration to infected tissues (37), antimicrobial resistant bacterial species (2, 25), and loss of tissue or organ function to the patient during treatment (12, 38). Challenges are augmented by the negative impact of treatment on patient quality of life (25), increased risk of bacterial infection upon hardware reimplantation (39), and the ability of *S. aureus* to evade the host immune system (30, 40).

Bacterial osteomyelitis has a progressively increasing incidence, and it is important to reduce morbidity and mortality to patients while concurrently reducing the burden on the healthcare system (11). Continued improvements in the understanding, diagnosis, and therapy of bacterial osteomyelitis are necessary to accomplish these goals. As a result of variable patient population, case presentation and disease management, clinical osteomyelitis research has proven difficult (38). A major step in achieving improved diagnostic and therapeutic methods lies within animal modeling of this disease. *In vivo* models facilitate groundbreaking research by allowing scientists to expand upon promising *in vitro* discoveries and utilize research findings to improve the lives of patients suffering from osteomyelitis. Ultimately, animal models promise to speed advances in modern medicine. The purpose of this review is to highlight a range of animal models used to study bacterial osteomyelitis. While it is not possible to present all of the features for each individual model, this review will emphasize the limitations and benefits of the most common animal models used to investigate the pathogenesis, diagnostic methods, and therapeutic strategies to better understand and combat bacterial osteomyelitis.

## Model Development

There are many approaches to inducing bacterial osteomyelitis in animal models. This review will focus on two main categories of bacterial osteomyelitis induction: surgical and hematogenous.

Authors have chosen to exclude detailed discussion of *in vivo* modeling of osteomyelitis secondary to DFU. There are reports of modeling bacterial infection with diabetic rodent strains, however, osteomyelitis resulting from DFU is a multifactorial, chronic condition and the complexity of modeling and translational healing differences raise concerns regarding reliable *in vivo* models (41–44). To surgically induce bacterial osteomyelitis in any species, there are a few necessary components. An injury to bone tissue (45) is required, and typically stems from mechanical trauma with or without the addition of a sclerosing agent (22, 46). A foreign body or medical device may be used to serve as a nidus for bacterial colonization (47). Bacterial inoculation is necessary and may be accomplished *via* direct administration of a bacterial inoculum (48), soaking of a foreign object or hardware in a bacterial suspension, creating a biofilm on a piece of hardware for implantation (49), or by intravenous (IV) administration of bacterial suspension (hematogenous seeding) (50). Many investigators choose to seal the bone defect, e.g. using sterile bone wax to ensure local containment of the bacteria and minimize undesired concomitant soft tissue infections (51). Induction of hematogenous bacterial osteomyelitis typically carries the advantage no required surgical manipulations or placement of foreign materials (52, 53). Hematogenous models are designed to closely mimic the acute hematogenous osteomyelitis that most commonly occurs in pediatric patients (52, 54).

Within these two categories, many differences exist in model design. Differences include the type of bone injury and surgical approach, bacterial strain and colony forming unit (CFU) count, administration vehicle and quantity of bacterial inoculum, as well as length of study and monitoring techniques. It is crucial to consider the bacterial species and strain that will be utilized in animal modeling. During initial model establishment, it is recommended to utilize a bacterial strain with well documented behavior within the chosen animal species. After confirming that osteomyelitis can be established in the selected model, the bacterial species, strain, dose, and even delivery vehicle may be altered to best accomplish the research objectives. On that token, investigators should consider the species-specificity, antimicrobial sensitivity profile, and clinical relevance of the chosen pathogen. These pillars of model development are highlighted by Laratta et al. (55) and commented on by Johansen et al. (56). Markers of success within model development typically include clinical manifestation of disease, evidence of osteomyelitis on histopathology, and positive bone cultures upon study completion. Most investigators elect to pulverize bone samples and perform bacterial culture from the pulverized samples. Confirmation of bacterial cultures using polymerase chain reaction (PCR) has become routine since the method was described in 1999 (54).

## Small Animal Models

### Mouse Models

#### Model Development

There are many surgical models of bacterial osteomyelitis performed in murine models. Models typically utilize long bones, although alternatives such as vertebral models are also reported (57). An extensive review of murine models, including the goal, method, and bacterial inoculum used in each study, was recently published by Guarch-Pérez et al. (58). One approach used by multiple investigators was described in 2008 as a model to assess intramedullary response to titanium particles (59). This surgical approach is accomplished by creating a medial parapatellar arthrotomy to access the femur. Once accessed, a defect extending to the medullary cavity of the femur is created. Kirschner wire (K-wire) is inserted into the femoral medullary canal and penetrated into the patellofemoral joint space. Bacterial inoculation occurs *via* direct application of a bacterial suspension and the surgical site is closed (48, 60, 61). This model was recently adapted and modified to model shoulder implant infections (62). In this study, investigators were able to reliably induce bacterial osteomyelitis using a bioluminescent strain of *S. aureus* and were able to track infection with radiographs and bioluminescent imaging (BLI). Another surgical approach that is utilized in various forms by many investigators is described well by Funao et al. (63). Much of this approach is similar to that described above; the distal portion of the femur is exposed surgically, and a 0.5 mm drill hole is created to expose the medullary canal of the femur. Rather than placing an implant, bioluminescent *S. aureus* is inoculated directly into the defect. The defect is then sealed with bone wax and the surgical site is closed. Another unique model of murine bacterial osteomyelitis is the hematogenous model described by Horst et al. (52). This model does not involve surgical manipulation or placement of foreign material. Instead, mice received one injection of *S. aureus* in phosphate-buffered saline (PBS) *via* the lateral tail vein. This model was created to closely mimic both acute and chronic hematogenous bacterial osteomyelitis and is unique in that it does not require additional bone injury. These approaches highlight the various methods available to induce bacterial osteomyelitis and the subtleties between the various models.

#### Insights Into Pathogenesis

While arguably each investigation into bacterial osteomyelitis provides information on pathogenesis, there are experiments designed to evaluate specific questions regarding the pathogenesis of bacterial osteomyelitis (64). One such experiment, described by De Mesy Bentley et al. (65), utilized two murine long bone infection models and captured groundbreaking transmission electron microscopy (TEM) images of *S. aureus* invading and residing within the osteocyte lacuno-canalicular network (OLCN) of live bone. *Staphylococcus aureus* cells are thought to be protected while within the canaliculi system, as immune cells are likely too large to successfully access this area of the body. Therefore, these findings offer insight into the ability of *S. aureus* to evade the host immune system and cause latent and recurrent osteomyelitis. Zoller et al. (40), established and utilized a murine model of bulk allograft infection to expand upon the findings of de Mesy Bentley et al. by investigating the mechanisms of immune system evasion by *S. aureus*, specifically microarchitecture of implant surfaces as a potential factor in increased bacterial colonization. *Staphylococcus aureus* was discovered within allograft cortical haversian canals and submicron canaliculi within the native mouse femur. Results indicated that bulk allograft implant material was more susceptible to bacterial infection even at low bacterial inoculums compared to stainless steel implants. This finding suggests that implant microarchitecture is incredibly important and may offer bacteria a submicron reservoir to evade clearance by the immune system. The work of Masters et al. (66) expanded upon these findings by investigating the role of *S. aureus* cell wall synthesis machinery and surface adhesins in OLCN invasion. The authors established a model of bacterial osteomyelitis by placing stainless steel pins inoculated with various mutant strains of *S. aureus* into the medial tibia of mice. Results showed significant changes in OLCN invasion, abscess formation and pathogenic bone loss with the deletions of penicillin binding protein 3 and 4 (PBP3, PBP4) and autolysin (Atl), indicating that cell wall synthesis machinery can modulate *S. aureus'* pathogenesis in osteomyelitis.

#### Improvements in Diagnostic Capabilities

While there are multiple reports of utilizing BLI and *in vivo* micro-CT in murine models (63, 67, 68), these reports are often geared toward improving the *in vivo* modeling system rather than improving diagnostic capabilities for clinical patients (63). Recently, however, Isogai et al. (69) performed plasma metabolome analysis in a model of murine osteomyelitis caused by *S. aureus* and identified 12 metabolites as candidate positive biomarkers and two candidate negative biomarkers for osteomyelitis. Novel plasma biomarkers are aimed to improve the early diagnosis of osteomyelitis. Improvement in the early diagnosis of osteomyelitis is of great interest, as there are currently many challenges in obtaining a swift and specific diagnosis in clinical patients.

#### Investigations Into Therapeutic Strategies

A major goal of *in vivo* osteomyelitis work is to evaluate novel treatment strategies and investigate potential efficacy for clinical use. There are many investigations focused on various combinations or applications of antibiotics for clearance of osteomyelitis (48, 70–72). Jørgensen et al. modeled the particularly challenging situation of biofilm presence upon orthopedic implants. They investigated the efficacy of rifampicin-containing combinations of antimicrobials compared with non-rifampicin-containing combinations of antimicrobials in reducing bacterial counts or clearing infection. Results indicated that combinations of antimicrobials that included rifampicin, as well as the combination of daptomycin and linezolid, were more effective in reducing bacterial burden than combinations not containing rifampicin (70). There are also many investigations into novel therapeutics (73–75). Wang et al. utilized a model of *S. aureus* hematogenous orthopedic implant infection to identify specific virulence factors to be translated into therapeutic targets. This work identified two key pathogenic factors, anti-α-toxin (AT) and anti-clumping factor A (ClfA) and demonstrated markedly improved efficacy in infection treatment utilizing human anti-AT/anti-ClfA combination therapy (50). Similarly, Yokogawa et al. (76) created a novel murine one-stage revision model of methicillin-resistant *S. aureus* (MRSA) implant-associated osteomyelitis. This model facilitated discovery of synergistic activity of vancomycin and anti-glucosaminidase (Gmd). Identification of alternative therapeutics is important, as medical device implantation continues to increase and antimicrobial resistance (AMR) is becoming increasingly prevalent.

#### Conclusion

Murine models are particularly helpful to researchers investigating bacterial osteomyelitis. Main attractions of the mouse model include the small size, economics, and genetic and molecular tools that are available to tailor murine strains and facilitate a wide array of investigations. Indeed, mouse strain selection is of paramount importance as strains contain significant differences from one another. Investigators should consider the primary research objective of the model to guide strain selection and ensure research objectives can be accomplished appropriately. This pillar of model development is highlighted nicely by Dworsky et al. (57). These advantages make mice attractive for investigations into pathogenesis and proof of concept models (58, 72). Also, mice allow for certain longitudinal monitoring techniques, such as BLI and *in vivo* microCT. Longitudinal monitoring is an asset that adds strength and clarity to data collection as individuals can be compared to themselves over multiple timepoints. While mice can mimic the human inflammatory response of osteomyelitis (58), their bone structure and bone remodeling process is less similar to humans than other animal models provide (77). As a result of the mouse's small size, complex and multi-stage surgical procedures are not impossible, but are challenging to perform. This small size also prohibits the investigation and translation of implants intended for human use. Additionally, serial blood collection is limited by volume and frequency. When considering the benefits and limitations of murine models, it can be concluded that mice are an excellent tool for early investigations from *in vitro* to *in vivo* modeling and proof of concept work.

### Rat Models

#### Model Development

Rats provide a variety of models that produce well-characterized and reliable bacterial osteomyelitis. Significant historical developments have previously been described (36, 64, 78, 79). Currently, the most popular rat models are of long bone osteomyelitis and most often utilize the tibia (17, 22, 80–83) or femur (84–90). Long bone models rely on mechanical trauma, placement of foreign bodies, or creation of fractures, all typically with concurrent sealing of the defect area with bone wax to contain bacterial inoculums and prevent concomitant soft tissue infection. Alternative models include mandibular models (91), vertebral models (92), joint prosthesis models (93), and hematogenous models (94, 95). Hematogenous models required additional surgical manipulations to successfully establish osteomyelitis. This may be a result of the rat's ability to respond to acute infection, which can rapidly clear peripheral infection and may complicate infection models (78). Although reports of rat osteomyelitis models exist, a comprehensive review of these models is lacking. In this review, we present a detailed summary of rat osteomyelitis models that were utilized to inform this review (Table 1).

**Table 1:** Rat models of osteomyelitis.

**Reference**	**Title**	**Sex, strain**	**Age, weight**	**Study endpoint(s)**	**Bacterial strain, inoculum size and volume**	**Inoculation method**	**Brief description of procedure**	**Evaluation methods**
*Rissing et al. (96)	Model of experimental chronic osteomyelitis in rats	Albino Sprague-Dawley	300–400 g	35 and 70 days	*Staphylococcus aureus* 52/52A/80 and OM-1. 3 × 10^6^ CFU/5 μl	Injection into intramedullary canal	Defect to tibial metaphysis, with medullary exposure, either *via* drill or needle. Application of sclerosing agent. Sealed with bone wax.	Histology, pathology, microbiology, radiographs, blood analyses
Spagnolo et al. 1993 (97)	Chronic *Staphylococcal* osteomyelitis: a new experimental rat model	Male, Wistar	250–350 g	30, 60, 90 and 180 days	*Staphylococcus aureus* (clinical isolate), 2 × 10^6^ CFU/5 μl	Injection into defect	Defects to tibial metaphyses bilaterally. Fibrin glue placed in defect. Sealed with bone wax	Radiographs, microbiology, histology, pathology
*Hienz et al. (95)	Development and characterization of a new model of hematogenous osteomyelitis in the rat	Female, Wistar	200 g	14 days	*Staphylococcus aureus* Phillips (clinical isolate), 1 ml of 5 × 10^4^-10^8^ CFU/ml	Intravenous injection *via* femoral vein	Drill defects to mandibular ramus and tibial metaphysis. Application of sclerosing agent.	Radiographs, microbiology, histology
Lucke et al. (22)	A new model of implant-related osteomyelitis in rats	Female, Sprague-Dawley	5 months	28 days	*Staphylococcus aureus* ATCC 49230, 10^2^, 10^3^, 10^6^ CFU/10 μl	Injection into intramedullary canal	Burr defect into tibial metaphysis, placement of K-wire	Radiographs, blood and serum analyses, microbiology, histology
*Fukushima et al. (81)	Establishment of rat model of acute *Staphylococcal* osteomyelitis: relationship between inoculation dose and development of osteomyelitis	Male, Wistar	200–270 g	7 days	*Staphylococcus aureus* BB – Bovine mastitis, 6 × 10–10^5^/5 μl	Injection into intramedullary canal	Drill defect into tibial metaphysis, sealed with bone wax	Microbiology, pathology, histology
Makinen et al. (51)	Comparison of ^18^F-FDG and ^68^Ga PET imaging in the assessment of experimental osteomyelitis due to *Staphylococcus aureus*	Male, Sprague-Dawley	380 g	2 weeks	*Staphylococcus aureus* 52/52A/80, 0.05 ml of 3 × 10^8^ CFU/ml	Injection into intramedullary canal	Drill defect into tibial metaphysis, application of sclerosing agent, sealed with bone wax	PET, pQCT, microbiology, histology, radiology
Bisland et al. (13)	Pre-clinical *in vitro* and *in vivo* studies to examine the potential use of photodynamic therapy in the treatment of osteomyelitis	Female, Sprague-Dawley	250–300 g	At least 14 days	*Staphylococcus aureus* Xen29, 10^6^ CFU/ml	*Via* biofilm coating on K-wire	Bilateral defects to tibial metaphyses with medullary cavity exposure. K-wire inserted into medullary cavity. Sclerosing agent applied shortly after. Sealed with bone wax	Fluoroscopy, BLI
Aktekin et al. (17)	A different perspective for radiological evaluation of experimental osteomyelitis	Female, Wistar albino	6 months, 250 g	3 and 6 weeks	*Staphylococcus aureus* ATCC 25923, 10^5^ CFU/0.05 ml	Injection into intramedullary canal	Tibial intramedullary aperture by 19G needle and application of sclerosing agent. Sealed with bone wax	Radiographs, CT, DEXA scans
Ofluoglu et al. (92)	Implant-related infection model in rat spine	Male, Sprague-Dawley	6 months, 300–350 g	15 days	*Staphylococcus aureus*, 10 μl of 10^2^, 10^3^, or 10^6^ CFU	Injection into surgical site	Reaming of junction between vertebral lamina and facet joint, placement of titanium microscrew.	Microbiology, histology
Robinson et al. (84)	Development of a Fracture osteomyelitis model in the rat femur	Male, Sprague-Dawley	250–300 g	3 weeks	*Staphylococcus aureus* (clinical isolate), 10^4^ CFU/50 μl	Injection into intramedullary canal	Defect to distal femur with medullary exposure. Stainless steel pin insertion. Sealed with bone wax	Radiographs, microbiology, histology
Vergidis et al. (98)	Treatment with linezolid or vancomycin in combination with rifampin is effective in an animal model of methicillin-resistant *Staphylococcus aureus* foreign body osteomyelitis	Male, Wistar	215–475 g	7 weeks	MRSA (clinical isolate IDRL 6169), 50 μl of 5 × 10^5^ CFU/ml	Injection into intramedullary canal	Drill defect into tibial metaphysis with medullary cavity exposure. Placement of wire into canal. Sealed with dental gypsum	Microbiology
Hamza et al. (10)	Intra-cellular *Staphylococcus aureus* alone causes infection *in vivo*	Male, Sprague-Dawley	400–450 g	3 weeks	*Staphylococcus aureus* ATCC 25923, 5 × 10^8^ CFU/ml	*Via* inclusion into osteoblasts (UMR-106) and application to fracture site, or osteoblast preparation with extracellular *Staphylococcus aureus* inoculum applied to fracture site	Mid-shaft femoral fracture created *via* custom device. Fracture stabilized with K-wire	Blood analyses, radiographs, microbiology
Sanchez et al. (85)	Effects of local delivery of D-amino acids from biofilm-dispersive scaffolds on infection in contaminated rat segmental defects	Sprague-Dawley	N/A	2 weeks	*Staphylococcus aureus* UAMS-1 and Xen36, 10^2^ CFU	*Via* soaked type I bovine collagen	6 mm segmental femoral defect, stabilized with polyacetyl plate and K-wires	Microbiology
Søe et al. (93)	A novel knee prosthesis model of implant-related osteomyelitis in rats	Male, Sprague-Dawley	6–9 weeks, 300 g	42 days	*Staphylococcus aureus* MN8 and UAMS-1, 10 μl of 10^2−5^ CFU	Injection into intramedullary canals	Non-constrained knee prosthesis	Radiographs, microbiology, histology, biochemical analysis
Fölsch et al. (86)	Coating with a novel gentamicinpalmitate formulation prevents implant-associated osteomyelitis induced by methicillin-susceptible *Staphylococcus aureus* in a rat model	Male, Sprague-Dawley	5 months	42 days	*Staphylococcus aureus* subsp. *aureus Rosenbach*, 10^2^ CFU	Injection into intramedullary canal	Reaming of femoral intramedullary cavity *via* a stifle approach. Placement of K-wire	Blood analyses, radiographs, microbiology
Stadelmann et al. (99)	*In vivo* microCT monitoring of osteomyelitis in a rat model	Female, Wistar	15 weeks, 276 g	28 days	*Staphylococcus aureus* (clinical isolate JAR 06.01.31), 3.3 × 10^7^ CFU/ml	*Via* soaking of experimental implant	Drill defect into tibial metaphysis. Placement of experimental implant	*In vivo* microCT, histology, microbiology
Vergidis et al. (100)	Comparative activities of vancomycin, tigecycline and rifampin in a rat model of methicillin-resistant *Staphylococcus aureus* osteomyelitis	Male, Wistar	250–350 g	7 and 9 weeks	MRSA (clinical isolate IDRL-6169), 5 × 10^5^ CFU/ml	Injection into intramedullary canal	Drill defect into tibial metaphysis with medullary cavity exposure. Placement of wire into canal. Sealed with dental gypsum	Microbiology
Avdeeva et al. (101)	Experimental simulation of traumatic osteomyelitis in rats	Male, Albino	200–250 g	21 days	*Staphylococcus aureus*	Injection into intramedullary canal	Defect to distal femoral metaphysis with thick needle	Blood analyses, histology
Fölsch et al. (102)	Systemic antibiotic therapy does not significantly improve outcome in a rat model of implant-associated osteomyelitis induced by Methicillin susceptible *Staphylococcus aureus*	Male, Sprague-Dawley	5 months	42 days	*Staphylococcus aureus* subsp. *aureus Rosenbach*, 10^2^ CFU	Injection into intramedullary canal	Reaming of femoral intramedullary cavity *via* a stifle approach. Placement of K-wire	Blood analyses, radiographs, microbiology
Harrasser et al. (82)	A new model of implant-related osteomyelitis in the metaphysis of rat tibiae	Male, Wistar	5 months, 350–400 g	42 days	*Staphylococcus aureus* ATCC 25923, 10^2^ or 10^3^ CFU/10 μl	Injection into intramedullary canal	Unicortical tibial metaphyseal defect with placement of experimental implant	Radiographs, microbiology, histology
Oh et al. (88)	Antibiotic-eluting hydrophilized PMMA bone cement with prolonged bactericidal effect for the treatment of osteomyelitis	Sprague-Dawley	250–300 g	4 and 8 weeks	*Staphylococcus aureus* (clinical isolate KCTC1621) 100 μl of 10^4^ CFU/ml	Injection into intramedullary canal	Defect to distal femur with medullary exposure. Sealed with bone wax	MicroCT, blood analysis
Park et al. (103)	Activity of tedizolid in methicillin-resistant *Staphylococcus aureus* experimental foreign body-associated osteomyelitis	Male, Wistar	250–350 g	7 weeks	MRSA (clinical isolate IDRL-6169), 50 μl of 10^6^ CFU/ml	Injection into intramedullary canal	Drill defect into tibial metaphysis with medullary cavity exposure. Placement of wire into canal. Sealed with dental gypsum	Microbiology
Hassani Besheli et al. (80)	Sustainable release of vancomycin from silk fibroin nanoparticles for treating severe bone infection in rat tibia osteomyelitis model	Male, Wistar	260–330 g	3 weeks	MRSA, ATCC 43300, 40 μl of 1–2 × 10^8^ CFU/ml	Injection into intramedullary canal	Burr defect into tibial metaphysis, placement of K-wire	Blood analysis, histology
Cui et al. (104)	Masquelet induced membrane technique for treatment of rat chronic osteomyelitis	Male, Sprague-Dawley	8 week, 190–220 g	20 weeks	*Staphylococcus aureus*, 0.3 ml	Injection into intramedullary canal	Modified blunt trauma method (101)	Blood analyses
Kussman et al. (105)	Dalbavancin for treatment of implant-related methicillin- resistant *Staphylococcus aureu*s osteomyelitis in an experimental rat model	Male, Sprague-Dawley	260–330 g	3 weeks	MRSA ATCC 43300, 40 μl of 1–2 × 10^8^ CFU/ml	Injection into intramedullary canal	Burr defect into tibial metaphysis, placement of K-wire	Blood analysis, histology
Melicherčík et al. (89)	Testing the efficacy of antimicrobial peptides in the topical treatment of induced osteomyelitis in rats	Male, Wistar	250 g	17 days	*Staphylococcus aureus* CNCTC 6271 (ATCC 43300; MRSA). 100 μl of 10^8^ CFU/ml	Injection into intramedullary canal	Reaming of femoral intramedullary cavity *via* a stifle approach	Radiographs
Neyisci et al. (83)	Treatment of implant-related methicillin- resistant *Staphylococcus aureus* osteomyelitis with vancomycin-loaded VK100 silicone cement: An experimental study in rats	Female, Sprague-Dawley	18–20 weeks	4 weeks	MRSA N315 (NBCI Taxonomy ID: 158879), 10^8^ CFU/ml	Injection into intramedullary canal	Reaming of tibial intramedullary canal with K-wire. Insertion of needle into canal. Sealed with bone wax. Implant removal at 2 weeks	Radiographs, microbiology, histology
Cobb et al. (106)	CRISPR-Cas9 modified bacteriophage for treatment of *Staphylococcus aureus* induced osteomyelitis and soft tissue infection	Female, Sprague-Dawley	13 weeks	8 days	*Staphylococcus aureus* ATCC 6538-GFP	*Via* soaked implant. Avg CFU: 5 × 10^4^	Bicortical drill defect to mid-femoral diaphysis. Placement of contaminated screws	Radiographs with fluorescent overlays, microbiology, histology, SEM
Jung et al. (87)	*In situ* gelling hydrogel with anti-bacterial activity and bone healing property for treatment of osteomyelitis	Sprague-Dawley	N/A	3 and 6 weeks	*Staphylococcus aureus*, 100 μl of 10^4^ CFU/ml	Injection into intramedullary canal	Defect to distal femur with medullary exposure. Sealed with bone wax	MicroCT, microbiology
Wu et al. (107)	Virulence of methicillin-resistant *Staphylococcus aureus* modulated by the YycFG two-component pathway in a rat model of osteomyelitis	Female, Sprague-Dawley	260–280 g	4 weeks	MRSA (clinical strain) and ASyycG over- expression MRSA clinical strain (ASyycG mutant). 40 μl of mid-exponential phase	Injection into intramedullary canal	Drill defect to antero-medial tibia with medullary cavity exposure	*In vivo* microCT, histology, SEM, rtPCR
Zhou et al. (108)	The synergistic therapeutic efficacy of vancomycin and omega-3 fatty acids T alleviates *Staphylococcus aureus*-induced osteomyelitis in rats	Male, Albino	180–200 g	At least 7 days	MRSA 1 × 10^6^ CFU/ml	Injection into intramedullary canal	Defect to tibial metaphysis, with medullary exposure, *via* dental burr. Reaming of medullary cavity with K-wire	Biochemical markers, histology, microbiology
Deng et al. (109)	Extracellular Vesicles: A potential biomarker for quick identification of infectious osteomyelitis	Male, Wistar	8–10 weeks, 300–350 g	At least 3 days	*Staphylococcus aureus, Staphylococcus epidermidis, Pseudomonas aeruginosa*, and *Escherichia coli* (clinical isolates), 100 μl of 10^8^ CFU/ml	Injection into intramedullary canal	Defect to tibial metaphysis, with medullary exposure, *via* needle. Needle tip indwelling within medullary canal. Sealed with bone wax	Serum extracellular vesicles
Sahukhal et al. (110)	The role of the msaABCR operon in implant-associated chronic osteomyelitis in *Staphylococcus aureus* USA300 LAC	Sprague-Dawley	250–300 g	4, 8 and 15 days	*Staphylococcus aureus* USA300 LAC, msaABCR mutant, and msaABCR complementation	*Via* biofilm coating on K-wire. Avg. CFU: 6.09 × 10^5^	K-wire pin insertion into tibial metaphysis	MicroCT, microbiology, histology, cytokine analysis
Qu et al. (90)	Zinc alloy-based bone internal fixation screw with antibacterial and anti-osteolytic properties	Male, Sprague-Dawley	3 months	3 and 6 weeks	MRSA ATCC 43300, 10^7^ CFU	*Via* soaked experimental implant	Defect between distal femoral condyles with medullary exposure. Contaminated implant placed. Sealed with bone wax.	Radiographs, microbiology, histology, blood analyses,
Sodnomi-Ish et al. (91)	Decompression effects on bone healing in rat mandible osteomyelitis	Male, Sprague-Dawley	8 week, 230 g	4 weeks	*Staphylococcus aureus* ATCC 29213, 20 μl of 10^7^ CFU/ml	Injection into defect	4 mm defect to mandibular ramus, sealed with fibrin glue	MicroCT, histology, immunohisto chemistry, blood analyses

*Asterisks denote papers deemed by the authors to be seminal to rat osteomyelitis modeling*.

#### Insights Into Pathogenesis

Similar to murine models, rat models can be utilized for investigations into pathogenesis. Rat models have facilitated valuable discoveries, including investigations of virulence factors associated with *S. aureus* biofilms and the ability of *S. aureus* to function as an intracellular pathogen. Biofilms are well recognized as a source of recalcitrant bacteria that can impair antibiotic treatment of osteomyelitis and cause persistent or recurrent osteomyelitis, particularly when orthopedic implants are in place (13, 31, 111). Two studies that have pursued the *in vivo* investigation of biofilm virulence factors and genetic components in rats include the investigation by Wu et al. (107), which demonstrated that overexpression of AS*yycG* led to a reduction in biofilm formation and *in vivo* pathogenicity of MRSA in a model of rat tibial osteomyelitis; as well as the investigation by Sahukhal et al. (110) who utilized a model of implant-associated osteomyelitis. This investigation demonstrated that deletion of the *msaABCR* operon of *S. aureus* (USA300 LAC) resulted in defective biofilm production and reduced severity of bacterial osteomyelitis. The capability of *S. aureus* to function as an intracellular pathogen is considered to be a mechanism of immune system evasion and a source of recurrent, persistent osteomyelitis (31) and is supported by *in vitro* evidence (112, 113). Based on that *in vitro* evidence, Hamza et al. investigated and confirmed the ability of purely intracellular *S. aureus* to induce osteomyelitis in a rat model (10).

#### Improvements in Diagnostic Capabilities

Similar to murine models, rat models have allowed for improvements in diagnostic or longitudinal monitoring capabilities in experimental models. Examples of these improvements include the findings of Stadelmann et al. (99), who demonstrated the use of *in vivo* microCT to longitudinally monitor bacterial osteomyelitis in a rat tibial model, thus offering a method to limit numbers of animals needed for experiments and to add strength to collected data. Also, Aktekin et al. evaluated the utility of available scoring systems for the radiographic evaluation of experimental osteomyelitis. Authors utilized a tibial model of osteomyelitis and evaluated serial radiographs throughout their study period, ultimately concluding that it is best to evaluate and report each radiograph individually, rather than appointing a numerical grade from a previously published grading scale (17). This is a valuable report for experimental studies, and with appropriate radiographic interpretation, is likely to add strength to radiograph assessments. An improvement to *in vivo* studies that holds potential to translate into human medicine is the investigation into various tracers for positron emission tomography (PET) to successfully image osteomyelitis and differentiate between bone infection and bone healing (51). The work investigating PET tracers indicated that Gallium-68 (^68^Ga), did not accumulate in healing bone, only infected bone. This work brings interest to the use of ^68^Ga and PET for clinical patients, although further work is needed to clarify use and safety concerns. Another interesting foray into improving diagnostics for clinical patients was completed by Deng et al. who described the potential use of extracellular vesicles (EVs) as a diagnostic marker for acute osteomyelitis (109).

#### Investigations Into Therapeutic Strategies

Rats are recognized to be more resilient than mice and therefore are well suited to investigations into therapeutic strategies, such as antibiotic trials. Indeed, there are many investigations into antibiotic therapies. These include therapeutic efficacy assessments of systemic antibiotics administered solo or in combination (98, 100, 103, 105), investigations of local antibiotic delivery systems (80, 83, 88) and antibiotics in combination with alternative therapies such as omega-3 fatty acid supplementation (108). There also are investigations into novel therapeutic strategies such as the use of photodynamic therapy (PDT) to treat contaminated orthopedic implants and minimize reliance on antibiotic therapy to clear implant associated bacterial osteomyelitis (13). Recently, Cobb et al. (106) investigated the feasibility of utilizing a bacteriophage to mitigate bacterial osteomyelitis, biofilm, and soft tissue infection.

#### Conclusion

Rat models are a valuable animal resource in the study of osteomyelitis. They provide similar benefits to mice, including small size, economics, ease of housing and handling, and well-characterized strains that provide appropriate uniformity and enable study of disease pathophysiology relevant to that seen in people (97). Rats have the ability to tolerate sustained, high dose antibiotic therapy (97). While larger than mice, rats remain too small for assessment of orthopedic hardware for human use, and multi-step revision procedures, although not impossible, remain challenging. Uniquely, the rat is one of few ideal species for modeling of mandibular osteomyelitis (91, 95) because of their size, anatomy, and general hardiness. Therefore, the strength of rat models lies within the ability to investigate pathogenesis and pursue initial investigations into therapeutic strategies to further understand *in vitro* data and gain *in vivo* knowledge prior to utilizing a larger animal model.

### Rabbit Models

#### Model Development

Rabbits provide many useful and reliable models of bacterial osteomyelitis. The systematic review by Reizner et al. (78) details significant historical developments and the review by Bottagisio et al. provides a thorough overview of model development and utility (114). Historically and currently, the most utilized models are long bone models, including tibial (115–121), femoral (49, 122–124), and radial (125–129). Alternative models such as joint prostheses (130, 131), mandibular defects (132), vertebral models (133–136) and implant infection *via* hematogenous seeding (137) exist. Induction of osteomyelitis among these various models can be accomplished *via* mechanical trauma, either defect (117) or fracture (49) creation and bacterial contamination with or without application of a sclerosing agent or foreign body placement (115), or through placement of contaminated implants (49, 129). Bone wax may be used to seal defect areas and prevent bacterial leakage and concomitant soft tissue infection (116). A benefit of rabbits compared to smaller models such as mice and rats is the improved ability to model chronic osteomyelitis (138) and perform revision procedures such as debridement (23, 116), which improves the capabilities of modeling human osteomyelitis and therapy. Rabbits offer a distinct advantage in studying bone disease because full segmental defects of the radius can be created without the need to stabilize the bone using orthopedic implants.

#### Insights Into Pathogenesis

Majority of reports into pathogenesis utilize well-characterized and reproducible rabbit models and are related to the capabilities of various bacterial species and strains (117, 121, 128) to induce osteomyelitis, as opposed to mechanistic work that more often is performed in murine and rat models. For example, Gahukamble et al. (117) describe an investigation into the abilities of *Staphylococcus lugdunensis* (*S. lugdunensis*) and *Propionibacterium acnes* (*P. acnes*) to establish osteomyelitis in a model that was previously characterized with a strain of *S. aureus* isolated from an infected human hip prosthesis (139). Results indicated that both organisms could induce osteomyelitis and described varying severity and clinical presentation. This work again emphasizes the importance of considering model development and bacterial strain selection during experimental design.

#### Improvements in Diagnostic Capabilities

Similar to murine and rodent models, there are studies aimed to improve the longitudinal monitoring of experimental osteomyelitis in rabbit models (21, 115) to improve utility of animal modeling and reduce required animal numbers. Odekerken et al., demonstrated that ^18^F-FDG micro-PET is a sensitive diagnostic tool for detecting early bone pathology, including early osteomyelitis (21), even in the presence of titanium implants (118). This method of imaging could differentiate between aseptic and infected bone as early as three weeks post-operatively and post-infection. Authors suggest that ^18^F-FDG PET carries potential as an early detector of clinical osteomyelitis cases, which is further confirmed by a retrospective analysis of clinical osteomyelitis cases performed by Wenter et al. (140). An important investigation geared toward improving available diagnostics was performed in a rabbit model of chronic osteomyelitis. In this study, the capability of PCR to return positive results was compared with traditional osteomyelitis diagnosis *via* radiographs and bacterial cultures of bone biopsies taken *via* different methods. Results indicated that PCR was a sensitive diagnostic tool and described techniques to determine species identification (23). It deserves recognition that while PCR is a strong tool to detect low bacterial burdens or metabolically inactive bacteria that may not yield positive bacterial culture, PCR results will not provide antibiotic susceptibility data. The described PCR techniques are useful for experimental models and also offer utility for clinical cases.

#### Investigations Into Therapeutic Strategies

Rabbits are widely utilized to test therapeutic strategies for the clearance of bacterial osteomyelitis. Rabbits are hindgut fermenters, which means that they may process oral antibiotics differently than humans (64). Nonetheless, rabbits have been widely utilized for evaluation of systemic and locally delivered antibiotic therapies (114, 123, 141). Rabbits also are a useful modeling system for evaluation of antibacterial coatings upon implants and local drug delivery systems (114, 129), as demonstrated by the use of silver ion doped calcium phosphate beads (120). There have also been investigations into alternative therapies for osteomyelitis, including the work performed by Kishor et al., investigating the use of bacteriophages to clear chronic osteomyelitis (142). In this study, *S. aureus* specific phages were purified, characterized, and utilized as a therapeutic in a model of acute and chronic femoral osteomyelitis. High doses of phage cocktail were found to be effective to clear *S. aureus* infection. This work presents an intriguing consideration for specific therapy of bacterial osteomyelitis. Another interesting study investigated the use of locally applied ozonated oxygen in a rabbit femoral model. While this treatment did not eliminate osteomyelitis, it did seem to lessen the clinical and radiographic markers of disease (122).

#### Conclusion

Rabbits fill a unique niche in *in vivo* osteomyelitis research. They are often utilized when the research goal involves assessment of orthopedic hardware or locally applied therapeutics and a small animal is needed, whether that need is dictated by animal housing limitations or by stage of research development. Rabbits provide a more relevant size to evaluate some human orthopedic implants, as well as an appropriate size to be maintained long-term so that revision procedures can be performed. Rabbits also provide a more similar immune system and long bone density to humans than mice and rats provide (143, 144). Despite these benefits, rabbit models are accompanied by more complex challenges including respiratory depression under anesthesia, hindgut fermentation, which impacts the ability to assess oral antibiotic therapies, and variation in bone healing response of young rabbits compared to humans. Most rabbit modeling should be performed in mature rabbits to maximize translation of results to clinical patients.

## Large Animal Models

### Pig Models

#### Model Development

Pigs are not as widely utilized to model bacterial osteomyelitis, but the models that are available are effective, well-characterized, and have seen logical progression. Studies may utilize either mini-pigs or commercial pigs. Perhaps the most widely utilized model of porcine osteomyelitis is a hematogenous model (56, 145–149). Alternative models include mandibular osteomyelitis (46, 150), tibial implant-related osteomyelitis (151–154), and traumatic tibial osteomyelitis (155). When the hematogenous model of osteomyelitis was initially introduced, an inoculum of *S. aureus* (S54F9) was administered IV through a lateral ear vein without any additional trauma. This IV inoculation resulted in acute, suppurative pneumonic and osteomyelitic lesions. Lesions of osteomyelitis were found primarily in the long bones, but also in the costochondral junctions of ribs (147). This model has been modified and is most frequently used by administering bacterial inoculums into the femoral artery (145, 146, 148, 149). Femoral artery inoculation is reliable in inducing osteomyelitis localized to the injected limb. This technique may produce concurrent soft tissue infections, injection site abscesses, and the degree of disease during the study may be variable (148, 156). However, this remains a strong technique for modeling acute hematogenous (juvenile) osteomyelitis.

#### Insights Into Pathogenesis

Pigs have not been utilized as widely as mice and rats to investigate pathogenesis of osteomyelitis, but there are a few interesting reports. One such study was carried out in a hematogenous model of osteomyelitis to determine the infection potential and disease characterization of three different strains of *S. aureus* (56). This work compared the typically utilized strain of porcine *S. aureus* (SF549) with two human strains of *S. aureus* (UAMS-1 and NCTC-8325-4). Results indicated that UAMS-1 and NCTC-8325-4 were less successful in establishing osteomyelitis than the porcine specific strain. Authors hypothesize that this may be due to increased host specificity, in contrast to rodent models, and that inoculation dose may play a role, which again brings attention to the importance of model and bacterial strain selection during experimental design. Additionally, an interesting discovery of biofilm within bone lesions shortly after infection was made and raises the concern that biofilms may form quite early on in disease. Jødal et al. investigated blood perfusion using [^15^O]water PET, and confirmed their hypothesis that blood perfusion would be increased in osteomyelitis-diseased bone as compared to healthy bone. While blood perfusion was increased in diseased bone as compared to healthy bone, blood perfusion was four-fold greater in areas of soft tissue infection than diseased bone (156).

#### Improvements in Diagnostic Capabilities

Afzelius et al. (149, 157) have made multiple investigations involving ideal tracing agents for diagnosing osteomyelitis. They investigated the use of more specific radiotracers, including: ^68^Ga-labeled DOTA-K-A9, DOTA-GSGK-A11, [^18^F]NaF, [^68^Ga]Ga Ubiquicidin, and [^68^Ga]Ga-DOTA-Siglec-9, and compared them to the use of [^18^F]FDG. This study demonstrated no accumulation of the more specific radiotracers, but positive accumulation of [^18^F]FDG (149). Investigators also compared [^99m^Tc]Interleukin-8 (IL-8) scintigraphy with [^18^F]FDG PET/CT in a hematogenous porcine model of osteomyelitis and found that [^99m^Tc]IL-8 was simple to prepare and use, and that it was capable of detecting 70% of lesions compared with 100% sensitivity of [^18^F]FDG PET/CT. This makes [^99m^Tc]IL-8 scintigraphy a promising candidate for further investigation for use in children, to decrease the radiation exposure, as compared to utilizing [^18^F]FDG PET/CT (157). Another interesting study was performed by Lüthje et al., who investigated the regulation of various acute phase proteins during osteomyelitis and found a significant pro-inflammatory local response to osteomyelitis, with limited systemic response. These findings confirm that osteomyelitis remains challenging to diagnose based on systemic findings and adds to the understanding that local investigation is necessary (153).

#### Investigations Into Therapeutic Strategies

Most porcine studies thus far have been accomplishing model development, pharmacokinetic work (152) and diagnostic methods. There is even one investigation into bone regeneration techniques in the face of osteomyelitis (46). Hill et al. (155) completed a study utilizing tibial implant-associated osteomyelitis and found that they could prevent osteomyelitis by administering combination antibiotic therapy every 6 h for 7 days. Jensen et al. (154) comment that pigs provide an ideal model for investigation into implant surface coatings, medical and surgical treatment regimes, and vaccination against *S. aureus*.

#### Conclusion

Pigs, particularly mini-pigs, offer many benefits, including size that is appropriate for complex or multi-stage procedures and for assessments of orthopedic hardware for human use. Porcine bone possesses similar fracture stress to human bone (158), hematogenous modeling creates a very similar situation to juvenile hematogenous osteomyelitis, and the gastrointestinal system of pigs is appropriate to receive oral antibiotics. There are many challenges when using pig models, including rapid growth and excessive mature body weight when utilizing commercial pigs (159), shorter long bones than found in people (154), the greater expense associated with a large animal model, variation in degree of disease manifestation, as well as a generally fractious demeanor. Porcine models are not currently as widely utilized as small animal models of osteomyelitis but provide an ideal model for the study of hematogenous osteomyelitis, offer great capabilities into investigation of imaging techniques, and are an area of interest for further development in the modeling of osteomyelitis. In general, commercial pigs are suitable for proof of concept and model development work, as they are less expensive than mini-pigs, but for longer-term studies and more appropriate translational work, mini-pigs should be utilized.

### Sheep Models

#### Model Development

Kaarsemaker et al. (160) initiated development of ovine models of osteomyelitis *via* creation of a tibial defect and subsequent bacterial inoculum injection into the medullary cavity of adult sheep. This study provided valuable information, including the ability to establish osteomyelitis in sheep and also the requirement for peri-operative systemic antibiotics to lessen the risk of fatal sepsis. Since then, a variety of long bone models have been developed, focused on the tibia (161, 162) or the femur (163), and often involving hardware infected with biofilm or planktonic bacteria (164) with or without revision procedures (162, 165). There remain a variety of techniques of creating bone injury, from unicortical defects and medullary canal inoculation (165) to osteotomies stabilized with experimental hardware (166). Recently, Moriarty et al. (162) established a model to replicate a failed two-stage revision procedure utilizing a MRSA infected intramedullary nail. This will likely be a valuable model to evaluate therapeutic strategies moving forward.

#### Investigations Into Therapeutic Strategies

Most investigations into therapeutics in ovine models have been centered upon experimental implants, systemic or local antibiotic therapies, and the ability to replicate the multi-stage revision procedures utilized in human medicine. There have been multiple investigations into local drug delivery devices to clear osteomyelitis. Boot et al. performed a multi-stage revision procedure and compared an injectable hydrogel impregnated with gentamicin and vancomycin to an antibiotic-loaded bone cement impregnated with gentamicin and vancomycin. Investigators were able to clear significantly more cases of osteomyelitis in the experimental hydrogel group, compared to the bone cement group, thereby presenting this material as a promising candidate for further exploration (165). Stewart et al. investigated another concept in local drug delivery by creating a vancomycin-modified titanium plate that demonstrated decreased clinical signs of infection, prevented biofilm formation and promoted bone healing in an infected tibial osteotomy model (166).

#### Conclusion

Currently, sheep are most often utilized for investigations into therapeutics utilizing long bone models. As such, sections regarding pathogenesis and diagnostic innovations were not included. Regardless, sheep are a valuable animal resource for the modeling of bacterial osteomyelitis, particularly focused on long bones. Sheep provide an ideal long bone size to perform complex procedures, replicate the treatment strategies utilized in clinical cases such as multiple revision procedures, and assess orthopedic hardware and devices for human use. Many characteristics of ovine bone are similar to that of humans, including torsional stiffness and osteogenesis (64), which adds to the strength of ovine modeling. Challenges associated with ovine modeling include the risk of sepsis, which may require peri-operative antibiotics, as well as the cost of housing and maintaining a large animal.

### Goat Models

#### Model Development

Most caprine models of osteomyelitis utilize the tibia, although models have variable approaches. Salgado et al. described a unicortical tibial defect with concurrent application of a sclerosing agent. *Staphylococcus aureus* was inoculated into the medullary canal and the defect was sealed with bone wax. In this model, goats received a perioperative dose of IV antibiotics. Induction of osteomyelitis was successful and no goats suffered from fatal sepsis (167). In an adaptation of this model, the sclerosing agent and perioperative antibiotics were omitted, and osteomyelitis was successfully induced, again with no reported sepsis (168). Other tibial models include the internal fixation of a tibial osteotomy (169) and percutaneous pin placement throughout the tibia (170). Through these investigations, researchers have also proposed histology scoring systems, to aid in the evaluation of model development (169).

#### Investigations Into Therapeutic Strategies

Similar to sheep, goats serve as viable translational models for investigations into therapeutic strategies. Wenke et al. utilized a similar model to that of Salgado et al. to investigate the efficacy of tobramycin-loaded calcium sulfate pellets compared to the efficacy of tobramycin-loaded antibiotic beads to treat bacterial osteomyelitis. Calcium sulfate and bone cement formulations loaded with tobramycin performed well, raising interest into the use of calcium sulfates for local drug delivery, as they do not require an additional procedure for removal (168). Tran et al. (169) investigated a silver-based antibacterial coating on intramedullary nails. In an experiment utilizing two goats, the goat that received the experimental implant displayed less severe signs of osteomyelitis than the control goat. An interesting experiment was performed to investigate the utility of a directly applied electric current to eliminate osteomyelitis over the course of 3 weeks. Authors found that electric currents were able to prevent signs of infection and suggest that this would be effective in clinical situations (170). Salgado et al. also reported an investigation of muscle vs. non-muscle flaps for reconstruction of defects and effective clearance of osteomyelitis. This study was designed as a result of discrepancies in the literature, with some reports of muscle flaps being superior and vice versa. This study found no difference between muscle and non-muscle flaps and re-emphasized that the most critical factor in treatment of bacterial osteomyelitis is thorough debridement (38).

#### Conclusion

Similar to sheep, goats possess great utility in modeling bacterial osteomyelitis, and this utility lies primarily within the size and composition of the caprine long bones, specifically the tibia. Long bone size and composition makes goats ideal for complex procedures and multi-stage surgeries. Goats provide an excellent model for assessment of orthopedic hardware intended for human use, as well as examination of local drug delivery devices and experimental coatings. Goats have not suffered from the reported sepsis that affected sheep when receiving intramedullary bacterial inoculation, which may aid researchers when selecting either sheep or goats as a model. Similar to any large animal model, goats are accompanied by greater costs than small animal models. As the majority of caprine modeling has been performed to either establish a reliable model or assess treatment options, the sections for pathogenesis and diagnostic investigations were omitted.

### Dog Models

#### Model Development

Canine models have been used in the past to model osteomyelitis, although today they are not widely utilized. Similar to caprine and ovine models, canine models have primarily utilized long bones, specifically the tibia (53, 171, 172) and the femur (47, 173, 174), although a vertebral model has also been described (175). Models vary in approach. Deysine et al. described an injection of bacterial inoculum into the tibial nutrient artery without any additional trauma. This approach was effective in establishing osteomyelitis, but also resulted in the loss of three dogs from septicemia (53). Most other models report bone trauma and bacterial inoculation of the medullary canal, whether that is by direct inoculation or placement of an infected implant (47, 171, 174). Khodaparast et al. had success in establishing osteomyelitis *via* application of a penetrating captive bolt device to the tibia of dogs to create an open fracture. This approach was selected in order to mimic traumatic osteomyelitis. This model involved the placement of microdialysis probes for sample collection, which is a valuable tool (172) when investigating the dynamics of local environments, whether that is physiologic dynamics or drug delivery profiles.

#### Investigations Into Pathogenesis

As described above, Khodaparast et al. (172) established a tibial fracture model of canine osteomyelitis and placed microdialysis probes with the goal of exploring the role of vascular endothelial growth factor (VEGF) as a rate-limiting step in wound healing. This was investigated by measuring VEGF mRNA levels in response to *S. aureus* osteomyelitis and *S. aureus* osteomyelitis treated with a rotational gastrocnemius muscle flap. The muscle flap was investigated because wound healing is accelerated in the presence of well-vascularized tissue. VEGF mRNA levels were found to be greater in the animals with osteomyelitis that received the rotational muscle flap as compared to those who did not. This finding suggests that type of surgical closure impacts specific biological signals and cellular pathways, and may add strength to the recommendation for utilizing muscle flaps for improved wound healing in reconstructive surgeries. Another investigation into pathogenesis was performed by Chen et al. (175) who aimed to investigate the presence, type, and origin of bacteria adjacent to metal implants utilized in the surgical management of pyogenic vertebral osteomyelitis. Investigators found that bacteria were retrieved not only from metal implants, but also from surrounding bone, despite the lack of radiographic signs of infection. These findings suggest that metallic implants are not necessarily the source of persistent or recurrent bacterial infection in vertebral osteomyelitis.

#### Investigations Into Therapeutic Strategies

Despite there being few reports, there are canine models of osteomyelitis that investigate treatment strategies. Two models focused on the prevention of osteomyelitis, and found that the placement of gentamicin impregnated bone cement could prevent the development of osteomyelitis in the experimental models (171, 173). Similarly, Huneault et al. (174) investigated the ability of cross-linked high amylose starch (CLHAS) implants loaded with ciprofloxacin to prevent and cure chronic femoral osteomyelitis. This study demonstrated strong preventative efficacy of the ciprofloxacin loaded implants, and also showed that ciprofloxacin loaded implants and oral ciprofloxacin had similar efficacy in clearing bacterial osteomyelitis.

#### Conclusion

Dogs provide strong models for long bone osteomyelitis. Benefits include appropriate size to perform complex and multi-stage procedures, bone composition and density that is most similar to humans out of the available species (158), temperament that is amenable to handling, as well as well-characterized anesthetic and imaging protocols. Despite these strengths, canine models are no longer frequently utilized for osteomyelitis research. Osteomyelitis research is terminal, and ethical concerns are raised when considering these companion animals as research models. Therefore, despite the provided benefits, it is unlikely that dogs will have a resurgence in popularity for osteomyelitis modeling.

## Conclusions and Future Directions

Through the currently available reports of advancements in the management and understanding of osteomyelitis that animal models have facilitated, it is clear that animal models are vital in osteomyelitis research. With the plethora of available species and approaches to model bacterial osteomyelitis, it is also clear that each species provides specific strengths and certain shortcomings, as is highlighted in this review. Based on current information, we suggest an approach where proof of concept work is performed in small mammal models, either a mouse or a rat model. Advanced pathogenesis investigations can also be carried out in small mammal models, either a mouse, rat, or rabbit model. Complex treatment strategies, whether local or systemic, are best suited for large animal models, either mini-pigs, sheep, or goats, to mimic the human response as closely as possible. Improvements to diagnostic procedures may be performed in a variety of models; initial investigations, especially into novel imaging techniques, are best suited for rodent models. Ideally, imaging techniques would be validated in large animal models before preclinical testing. Regardless of the specific indication and utility, the knowledge we gain from animal models of osteomyelitis is an essential asset to the understanding, diagnosis and treatment of bacterial osteomyelitis, and animal modeling is a crucial step toward improving the lives of patients suffering from this life-altering disease.

## Author Contributions

CB: conceptualization, writing, and editing. DA: conceptualization, editing, and supervision. All authors contributed to the article and approved the submitted version.

## Conflict of Interest

The authors declare that the research was conducted in the absence of any commercial or financial relationships that could be construed as a potential conflict of interest.

## Publisher's Note

All claims expressed in this article are solely those of the authors and do not necessarily represent those of their affiliated organizations, or those of the publisher, the editors and the reviewers. Any product that may be evaluated in this article, or claim that may be made by its manufacturer, is not guaranteed or endorsed by the publisher.
